# Alta Mortalidade por Infarto Agudo do Miocárdio na América Latina e Caribe: Defendendo a Implementação de Linha de Cuidado no Brasil

**DOI:** 10.36660/abc.20220825

**Published:** 2022-11-23

**Affiliations:** 1 Universidade Federal de Minas Gerais Departamento de Clínica Médica Belo Horizonte MG Brasil Departamento de Clínica Médica – Universidade Federal de Minas Gerais, Belo Horizonte, MG – Brasil; 2 Hospital das Clínicas da Universidade Federal de Minas Gerais Serviço de Cardiologia e Cirurgia Cardiovascular Belo Horizonte MG Brasil Serviço de Cardiologia e Cirurgia Cardiovascular – Hospital das Clínicas da Universidade Federal de Minas Gerais, Belo Horizonte, MG – Brasil

**Keywords:** Infarto do Miocárdio, Epidemiologia, Mortalidade, Síndrome Coronariana Aguda, Políticas de Saúde Pública

A doença isquêmica do coração (DIC) é a principal causa de morte no mundo, e enquanto em países de alta renda (PAR) foram observados declínios substanciais nas taxas de mortalidade por DIC nas últimas décadas, o mesmo não ocorreu em países de baixa e média renda (PBMR).^1^ O evento final na cadeia da DIC é o infarto agudo do miocárdio (IAM), que pode ser classificado com base no eletrocardiograma em IAM com supradesnivelamento do segmento ST (IAMCSST) e IAM sem supradesnivelamento do segmento ST (IAMSSST) – o primeiro apresentando maior letalidade.^2^ As taxas de mortalidade para ambas as apresentações de IAM podem ser reduzidas pelo diagnóstico e tratamento rapidamente instituídos e de acordo de acordo com as diretrizes atuais, incluindo terapia de reperfusão para IAMCSST. Nos PAR, como os EUA, a mortalidade intra-hospitalar para IAMCSST variou de 3,5% para indivíduos que receberam angioplastia coronária percutânea primária a 14,9% para aqueles que não receberam reperfusão, enquanto em países europeus, foi relatada mortalidade tão baixa quanto 2,5%.^3,4^

Neste número da Revista, o artigo “Mortalidade Hospitalar Por Infarto do Miocárdio na América Latina e no Caribe: Revisão Sistemática e Metanálise” traz dados contemporâneos sobre a mortalidade hospitalar por IAM em LMIC da América Latina e Caribe de 2000 a 2020.^5^ Utilizando metodologia apropriada, os autores realizaram uma metanálise de dados de 38 estudos, principalmente realizados no Brasil, Cuba e Argentina: 35 para IAMCSST com 28.878 indivíduos, e 9 para IAMSSST com 2.377 indivíduos. A análise conjunta demonstrou que a mortalidade hospitalar por IAMCSST foi de 9,9% (IC 95%: 9,1–10,7), com heterogeneidade moderada a alta (*I*^2^=74%). O Chile apresentou a menor mortalidade (8,5%; IC 95%: 5,3-13,5) e a Colômbia a maior (15%; IC 95%: 10,1-21,7), com o Brasil apresentando mortalidade de 9,6% (IC 95%: 8,3 −11,0); no entanto, não foi encontrada diferença estatística entre os países (p=0,47). Para NSTEMI, a mortalidade intra-hospitalar foi de 7,2% (IC 95%: 5,5 – 9,3), também com heterogeneidade moderada a alta (*I*^2^ = 63%), explicada (*I*^2^ = 0%) pela exclusão de um estudo “outlier”.^5^

Os resultados descritos acima são robustos porque a redução da mortalidade por IAMCSST foi associada a fatores conhecidos que melhoram os desfechos, e que também explicam (sujeito: fatores) em parte a heterogeneidade: maior taxa de reperfusão (coeficiente=-0,009, IC 95%: −0,013 a −0,006, p<0,001) e avanços temporais no tratamento, com maior mortalidade em 2000-2009 em relação a 2010-2020 (coeficiente=-0,14, IC 95%: −0,27 a −0,02, p=0,047).^2^ No entanto, deve-se reconhecer que a mortalidade intra-hospitalar relatada é provavelmente menor do que os números reais, pois os estudos incluídos foram realizados em unidades de saúde mais organizadas, onde os pacientes podem ter tido melhor acesso ao tratamento do que a população total que apresentou IAM na região.

Ainda assim, as lacunas na mortalidade intra-hospitalar por IAMCSST entre PAR e América Latina/Caribe reforçam a possibilidade de reduzir a mortalidade por IAM em PBMR, proporcionando melhor acesso aos cuidados. Ao integrar e organizar estabelecimentos e profissionais de saúde em uma região, linhas de cuidado ao IAM trazem melhor acesso à reperfusão, às medicações baseadas em evidências e aos cuidados intensivos, levando a melhores resultados e otimização de recursos.^6^

No contexto brasileiro, a DIC é a primeira causa de mortalidade, respondendo por 12% de todas as mortes.^7^ As internações por IAM aumentaram 54% de 2008 a 2019 em hospitais públicos – com 12,9% de mortalidade intra-hospitalar em 2019 –, tornando o IAM um importante problema de saúde pública.^7^ Vale ressaltar que as disparidades no atendimento do indivíduo com IAM ocorrem dentro do país: em um registro de 4.782 pacientes de hospitais públicos e privados selecionados, a mortalidade intra-hospitalar foi de 3,4%, mas maior em hospitais públicos.^8^ Mortalidade maior também foi evidenciada em uma cidade brasileira na rede pública (19,5%) em relação aos hospitais privados (4,8%).^9^

Dessa forma, a implementação da linha de cuidado ao IAM no Brasil é fundamental para reduzir a mortalidade por IAM, na perspectiva da saúde pública. Em 2011, o Ministério da Saúde lançou a Portaria 2.994 para promover a organização da linha de cuidado ao IAM. Embora as experiências iniciais tenham reduzido com sucesso a mortalidade intra-hospitalar (4-6% das reduções absolutas), elas ficaram restritas a algumas áreas geográficas.^10–12^ Em 2021, os componentes pré-hospitalares da linha de cuidado também foram regulamentados pela Portaria 2.777, que inclui o uso de telemedicina para análise de ECG e suporte clínico, além de trombólise pré-hospitalar.

Muitos desafios para a implementação da linha de cuidado ao IAM foram descritos, particularmente para PBMR. Esses desafios relacionam-se ao diagnóstico tardio, às estratégias de encaminhamento e/ou infraestrutura de saúde inadequadas, ao financiamento insuficiente e aos atrasos na procura de atendimento.^6^ Suporte por telemedicina para diagnóstico eletrocardiográfico e atendimento clínico, organização de centros de referência, atendimento pré-hospitalar aprimorado - incluindo trombólise pré-hospitalar - e campanhas públicas sobre sintomas de IAM são estratégias para superar essas barreiras.^6,13^ Uma meta-análise recente mostrou que a inclusão da telecardiologia como parte das linhas de cuidado ao IAM foi associada a uma redução de 37% na mortalidade.^13^ Além disso, o envolvimento de diferentes partes interessadas, incluindo formuladores de políticas de saúde e profissionais de saúde pré-hospitalares e hospitalares, é a outra parte essencial para uma estratégia de implementação bem-sucedida.^6^

Apoiando-se nos princípios fundamentais do SUS de universalidade e equidade, a extensão da linha de cuidado ao IAM para todos os municípios brasileiros urge se quisermos reduzir a mortalidade por IAM, principal causa de morte em nosso país.
