# Supplementary material for: Alta Mortalidade por Infarto Agudo do Miocárdio na América Latina e Caribe: Defendendo a Implementação de Linha de Cuidado no Brasil
Source: Arq Bras Cardiol. 2022 Nov 23;119(6):979–80. [Article in Portuguese] doi: 10.36660/abc.20220825 (PMC9814805; doi:10.36660/abc.20220825)
Supplement: Translation

# Translation: High Mortality for Myocardial Infarction in Latin America and the Caribbean: Making the Case for Systems of Care Implementation in Brazil

**Affiliations:** 1 Belo Horizonte MG Brazil Departamento de Clínica Médica – Universidade Federal de Minas Gerais, Belo Horizonte, MG – Brazil; 2 Belo Horizonte MG Brazil Serviço de Cardiologia e Cirurgia Cardiovascular – Hospital das Clínicas da Universidade Federal de Minas Gerais, Belo Horizonte, MG – Brazil

**Keywords:** Myocardial Infarction, Epidemiology, Mortality, Acute Coronary Syndrome, Public Health Policy

Ischemic heart disease (IHD) is the leading cause of death worldwide, and while in high-income countries (HIC), substantial declines in mortality rates for IHD were observed in the last decades, the same did not occur in low- and middle-income countries (LMIC).^1^ The final event in the chain of IHD is myocardial infarction (MI), which can be classified based on the electrocardiogram in ST-segment elevation MI (STEMI), and non-ST-segment elevation MI (NSTEMI) - the first having higher lethality.^2^ Mortality rates for both MI presentations can be reduced by timely diagnosis and treatment according to current guidelines, including reperfusion therapy for STEMI.^3^ In HIC, such as the US, STEMI in-hospital mortality varied from 3.5% for individuals receiving primary percutaneous coronary angioplasty to 14.9% for those receiving no reperfusion, while in European countries, mortality as low as 2.5% has been reported.^3,4^

In this issue of the Journal, the article “Hospital Mortality from Myocardial Infarction in Latin America and the Caribbean: Systematic Review and Meta-Analysis” brings a welcome addition regarding MI in-hospital mortality in LMIC from Latin America and the Caribbean from 2000 to 2020.^5^ Using sound methodology, the authors made a meta-analysis of data from 38 studies, mostly conducted in Brazil, Cuba, and Argentina: 35 for STEMI with 28,878 individuals, and 9 for NSTEMI with 2,377 individuals. Pooled analysis demonstrated that in-hospital mortality for STEMI was 9.9% (95%CI: 9.1–10.7), with moderate to high heterogeneity (*I*^2^=74%). Chile had the lowest mortality (8.5%; 95%CI: 5.3-13.5), and Colombia the highest (15%; 95%CI: 10.1-21.7), with Brazil showing a mortality of 9.6% (95%CI: 8.3-11.0); however, no statistical difference was found among countries (p=0.47). For NSTEMI, in-hospital mortality was 7.2% (95% CI: 5.5 – 9.3), also with moderate to high heterogeneity (*I*^2^ = 63%), explained (*I*^2^=0%) by the exclusion of one outlier study.^5^

The results above described are robust because mortality reduction for STEMI was associated with known factors that improve outcomes, which also partly explained the heterogeneity: higher reperfusion rate (coefficient=-0.009, 95% CI: −0.013 to −0.006, p<0.001) and temporal advances in treatment, with higher mortality in 2000-2009 compared to 2010-2020 (coefficient=-0.14, 95% CI: −0.27 to −0.02, p=0.047).^2^ However, it should be acknowledged that the in-hospital mortality reported is probably lower than the actual numbers, as included studies were conducted in more organized health facilities, where patients may have had better access to treatment than the total MI population of the region.

Still, the gaps in STEMI in-hospital mortality between HIC and Latin America/Caribbean reinforces the possibility of reducing MI mortality in LMIC by providing better access to care. By integrating and organizing health facilities and providers in a region, MI care systems bring better access to reperfusion, evidence-based medication, and intensive care, leading to improved outcomes and optimizing resources.^6^

In the Brazilian context, IHD is the first cause of mortality, accounting for 12% of all deaths.^7^ Hospitalizations due to MI rose 54% from 2008 to 2019 in public hospitals - with 12.9% in-hospital mortality in 2019, making MI a major public health issue.^7^ Of note, disparities in MI care occur within the country: in a registry of 4782 patients from selected public and private hospitals, in-hospital mortality was 3.4%, but higher in public hospitals.^8^ Higher mortality was also shown in a Brazilian city in public (19.5%) compared to private hospitals (4.8%).^9^

As such, implementing the MI system of care in Brazil from the public health perspective is fundamental to reduce MI mortality. In 2011, the Ministry of Health launched the ordinance 2.994 to promote the organizations of MI systems of care. While initial experiences have successfully reduced in-hospital mortality (4-6% of absolute reduce), they have been restricted to some geographical areas.^10–12^ In 2021, pre-hospital components of the system were also regulated in the ordinance 2.777, which includes the use of telemedicine for the use of telemedicine for ECG analysis and clinical support, and pre-hospital thrombolysis.

Many challenges for implementing the MI systems of care have been described. They relate to late diagnosis, inadequate referral strategies and/or health infrastructure, insufficient funding, and delays in seeking care.^6^ Telemedicine support for ECG diagnosis and clinical care, organization of referral hubs, enhanced pre-hospital care – including pre-hospital thrombolysis – and public campaigns about MI symptoms are strategies to overcome these barriers.^6,13^ A recent meta-analysis showed that including telecardiology as part of MI systems of care was associated with a 37% reduction in mortality.^13^ Moreover, the involvement of different stakeholders, including health policymakers and pre-hospital and hospital healthcare providers, is another essential part of a successful implementation strategy.^6^

Laying in the fundamental principles of SUS of universality and equity, the extension of the MI system of care across every Brazilian municipality urges if we want to reduce mortality by MI, the number one cause of death in our country.
